# Analysis of the efficacy and safety of intravenous injection combined with nebulized inhalation of polymyxin E sodium methanesulfonate in the treatment of carbapenem resistant gram-negative bacterial infections in the lungs

**DOI:** 10.3389/fcimb.2026.1821093

**Published:** 2026-06-19

**Authors:** Yun Ma, Yanrui Zhang, Xiaowei Wang, Xin Zheng, Meng Xia, Qianluo Zhang

**Affiliations:** 1 Department of Pharmacy, The Eighth Hospital of Xi’an, Xi’an, China; 2 Department of Pharmacy, Dianjiang People’s Hospital of Chongqing, Chongqing, China

**Keywords:** biomarkers, carbapenem-resistant gram-negative pneumonia, multidrug-resistant infections, nebulized antibiotics, polymyxin E, ventilator-associated pneumonia

## Abstract

**Background:**

Carbapenem-resistant Gram-negative (CR-GN) pneumonia remains a major therapeutic challenge due to limited effective treatment options and high morbidity. Polymyxin E sodium methanesulfonate (colistin), despite concerns of nephrotoxicity, continues to serve as a last-resort agent. The adjunctive use of nebulized polymyxin may enhance local drug delivery and improve outcomes.

**Objective:**

To evaluate the clinical efficacy, microbiological clearance, radiological response, and safety of combined intravenous and nebulized polymyxin E in patients with CR-GN pneumonia, and to identify early predictors of treatment response.

**Methods:**

This retrospective, single-center observational study included 120 adult patients with microbiologically confirmed CR-GN pneumonia treated between January 2022 and December 2024. All patients received intravenous polymyxin E along with nebulized polymyxin E (50 mg twice daily). Clinical parameters, laboratory markers, radiological findings, and adverse events were assessed at baseline (Day 0), Day 5, and Day 10–14. Multivariate logistic regression and receiver operating characteristic (ROC) curve analysis were performed to identify predictors of poor outcomes.

**Results:**

Significant improvements were observed across clinical parameters, including reduction in temperature (38.6 °C to 36.9 °C), respiratory rate, and improvement in oxygenation (SpO_2_: 90.2% to 96.1%; PaO_2_/FiO_2_: 205.4 to 326.9; p < 0.01). Inflammatory markers, including C-reactive protein and procalcitonin, showed a marked decline over time. Microbiological clearance was achieved in 73.3% of patients, with early culture conversion observed in nearly half by Day 5. Radiological improvement ≥50% was noted in 79.2% of cases. Nephrotoxicity occurred in 15% of patients, bronchospasm in 7.5%, and treatment discontinuation in 3.3%. ROC analysis identified procalcitonin >1.2 ng/mL, CRP >45 mg/L, and PaO_2_/FiO_2_ <250 as significant predictors of poor clinical outcomes.

**Conclusion:**

Combined intravenous and nebulized polymyxin E therapy was associated with favorable clinical and microbiological outcomes in patients with CR-GN pneumonia, with an acceptable safety profile. Early biomarkers such as procalcitonin, CRP, and oxygenation indices may help guide risk stratification and optimize treatment strategies in critically ill patients.

## Introduction

The global rise of antimicrobial resistance (AMR) has emerged as one of the most critical threats to modern healthcare systems, significantly affecting infection control, patient outcomes, and healthcare costs. This challenge is particularly pronounced among Gram-negative bacterial pathogens, which possess a remarkable ability to acquire and disseminate resistance determinants through mechanisms such as horizontal gene transfer. As a result, infections caused by these organisms frequently exhibit resistance to multiple first-line and second-line antibiotics, leading to a narrowing of therapeutic options and the re-emergence of difficult-to-treat infections that were once considered manageable (Lu et al., 2022). Among these, carbapenem-resistant Gram-negative bacteria (CR-GNB), including Klebsiella pneumoniae, Acinetobacter baumannii, and Pseudomonas aeruginosa, have become especially concerning in hospital settings, particularly in patients with hospital-acquired pneumonia (HAP) and ventilator-associated pneumonia (VAP). The limited development of new antimicrobial agents in recent decades has further compounded this problem, prompting renewed interest in older antibiotics such as polymyxins. Polymyxin B and polymyxin E (colistin), initially introduced in the 1950s, have re-emerged as last-resort therapies for multidrug-resistant (MDR) and extensively drug-resistant (XDR) infections despite their well-documented toxicity profiles (Karaiskos et al., 2023; Ansems et al, 2024; Zhou et al., 2024). Polymyxin E is a cationic polypeptide antibiotic derived from Bacillus polymyxa that exerts bactericidal activity by disrupting the integrity of the bacterial outer membrane through interaction with lipopolysaccharides. This unique mechanism distinguishes it from other antibiotic classes such as β-lactams and aminoglycosides and contributes to its retained efficacy against resistant Gram-negative pathogens (Ben Lakhal et al., 2021). However, its clinical utility is often limited by adverse effects, particularly nephrotoxicity, which may occur in up to 50% of patients, as well as less frequent neurotoxicity manifestations such as dizziness, paresthesia, and neuromuscular blockade (Chang et al., 2021) Monsel et al., 2021. These safety concerns have necessitated the exploration of optimized delivery strategies to enhance efficacy while minimizing systemic toxicity. In this context, nebulized administration of polymyxin E has gained increasing attention as an adjunct to intravenous therapy, particularly for lower respiratory tract infections. The inhalational route allows direct delivery of the drug to the site of infection, achieving higher local concentrations in pulmonary tissues while potentially reducing systemic exposure. This targeted approach may be particularly beneficial in VAP, where biofilm formation and airway secretions can limit the effectiveness of systemically administered antibiotics (El-Sayed Ahmed et al., 2020; Wagenlehner et al., 2021). Several studies have suggested that the combination of intravenous and nebulized polymyxin therapy may improve microbiological eradication, accelerate clinical recovery, and reduce the duration of mechanical ventilation (Tsuji et al., 2019).

Despite these promising findings, important challenges remain. Variability in nebulization techniques, drug stability, particle size distribution, and ventilator circuit compatibility may influence drug delivery and clinical outcomes (Hasan et al., 2021). In addition, there is a lack of standardized dosing regimens for inhaled polymyxins, with some studies advocating fixed dosing while others recommend individualized approaches based on pharmacokinetic modeling or therapeutic drug monitoring (Lyu et al., 2020; Zheng et al., 2020; Almangour et al., 2021; Hasan et al., 2021). Furthermore, heterogeneity in study designs and patient populations has led to inconsistent results across the literature, limiting the generalizability of existing evidence (Kellum and Lameire, 2013; Choe et al., 2019; Ahn et al., 2020; Zhou et al., 2021; Lin et al., 2022; Wu et al., 2023). Safety considerations also remain a key concern. While nebulized administration may reduce systemic toxicity, particularly nephrotoxicity, local adverse effects such as bronchospasm and airway irritation have been reported, especially with higher doses (Matijašević et al., 2018; Jain et al., 2021; Fumagalli et al., 2022; Kalil and Cawcutt, 2022; Liu et al., 2022; Lu and Mao, 2023; Shi et al., 2023; Zhang et al., 2023). Therefore, a careful evaluation of both efficacy and safety is essential when considering combination therapy with intravenous and inhaled polymyxin E. The clinical burden of CR-GNB pneumonia has been further exacerbated in recent years, particularly during the COVID-19 pandemic. Prolonged ICU stays, increased use of mechanical ventilation, and extensive antibiotic exposure have contributed to a rise in secondary bacterial infections and multidrug resistance. Several studies have reported an increased incidence of HAP and VAP caused by CR-GNB in critically ill COVID-19 patients, highlighting the urgent need for effective and optimized treatment strategies in this setting (Goyal et al., 2020; Grasselli et al., 2021; Howatt et al., 2021; Brink et al., 2022; Vacheron et al., 2022; Feng et al., 2023).

Although previous studies have evaluated the clinical utility of nebulized polymyxin, there remains a relative lack of real-world evidence integrating clinical, microbiological, radiological, and laboratory outcomes within a single framework. Moreover, limited attention has been given to the role of early biomarkers such as procalcitonin, C-reactive protein (CRP), and oxygenation indices in predicting treatment response and guiding therapeutic decisions in patients with CR-GNB pneumonia. Therefore, the present study aims to evaluate the clinical efficacy and safety of combined intravenous and nebulized polymyxin E sodium methanesulfonate in patients with CR-GNB pneumonia in a real-world clinical setting. In addition, this study seeks to identify early predictors of treatment response using clinical and laboratory parameters, thereby contributing to more individualized and evidence-based management of critically ill patients. Radiological and microbiological assessments were incorporated to provide a comprehensive evaluation of treatment outcomes, with radiological improvement serving as a surrogate marker of pulmonary recovery and microbiological clearance confirming pathogen eradication (Grégoire et al., 2017; Karaiskos et al., 2019).

## Materials and methods

### Study design and setting

This retrospective, observational, single-center, single-arm study was conducted in the Department of Pulmonary Medicine in collaboration with the Departments of Microbiology, Radiology, and Critical Care Medicine at a tertiary care teaching hospital from January 2022 to December 2024. The study aimed to evaluate the effectiveness and safety of combined intravenous and nebulized polymyxin E sodium methanesulfonate in the treatment of carbapenem-resistant Gram-negative (CR-GN) lower respiratory tract infections.

A total of 120 adult patients (≥18 years) with microbiologically confirmed CR-GN pulmonary infections were consecutively included. Ethical approval was obtained from the Institutional Ethics Committee (Approval No.:2025-K-051), and written informed consent was obtained from all participants prior to inclusion.

### Study population

#### Inclusion criteria

Patients were eligible if they met the following criteria:

- Age ≥18 years.
- Confirmed diagnosis of CR-GN pulmonary infection (hospital-acquired pneumonia [HAP] or ventilator-associated pneumonia [VAP]).
- Microbiological confirmation via sputum, endotracheal aspirate, or bronchoalveolar lavage.
- Pathogen susceptibility to polymyxin E [minimum inhibitory concentration (MIC) ≤2 µg/mL].
- Requirement for polymyxin E therapy due to resistance to other antibiotics.

#### Exclusion criteria

Patients were excluded if they had:

- Known hypersensitivity to polymyxin antibiotics.
- Pregnancy or lactation.
- Chronic kidney disease requiring dialysis.
- Co-infection with active fungal, mycobacterial, or viral pulmonary infections.
- Immunocompromised status (e.g., HIV with CD4 <200 cells/mm³, organ transplant recipients).

### Treatment protocol

All patients received intravenous polymyxin E sodium methanesulfonate in accordance with institutional treatment protocols. In addition, nebulized polymyxin E was administered as adjunctive therapy to achieve higher local drug concentrations in the lungs.

Nebulized polymyxin E was administered at a dose of [e.g., 50 mg twice daily], reconstituted according to manufacturer recommendations, and delivered via a [jet/mesh] nebulizer system integrated into the ventilator circuit for mechanically ventilated patients or via standard inhalation devices for non-ventilated patients.

### Dose modification in renal impairment

In patients with impaired renal function (creatinine clearance <50 mL/min), dose adjustments were made to prevent drug accumulation. Maintenance doses were reduced by 25–50%, and dosing intervals were extended where necessary. Renal function was monitored daily using serum creatinine and urine output, and dose adjustments were guided by institutional nephrology recommendations.

### Concomitant antibiotic therapy

Combination antibiotic therapy was administered in selected patients based on antimicrobial susceptibility patterns and clinical judgment. Commonly used agents included meropenem, tigecycline, and amikacin, particularly in infections caused by multidrug-resistant organisms such as Klebsiella pneumoniae and Acinetobacter baumannii. These regimens were guided by antimicrobial stewardship protocols, and their potential confounding effects were considered during analysis.

### Monitoring during therapy

All patients were monitored daily for both treatment efficacy and adverse events. Clinical parameters, including body temperature, respiratory rate, oxygen saturation (SpO_2_), and PaO_2_/FiO_2_ ratio, were recorded regularly. Laboratory investigations included complete blood count, renal and liver function tests, arterial blood gas analysis, procalcitonin (PCT), and C-reactive protein (CRP).

Follow-up microbiological samples (sputum or endotracheal aspirate) were collected on Day 5 or earlier in case of clinical deterioration. Adverse events, including nephrotoxicity, neurotoxicity, and bronchospasm, were recorded and graded according to the Common Terminology Criteria for Adverse Events (CTCAE) version 5.0.

### Outcome measures

#### Clinical parameters

Clinical improvement was assessed using vital signs, oxygenation indices, and symptom evaluation. Body temperature was recorded daily to assess fever resolution. Respiratory rate and oxygen saturation were monitored regularly. Dyspnea was evaluated using the Modified Medical Research Council (mMRC) scale at baseline, mid-treatment, and end of therapy. Changes in cough and sputum production were also documented. The PaO_2_/FiO_2_ ratio was calculated at baseline (Day 0), Day 5, and Day 10–14.

#### Laboratory parameters

Laboratory assessments were performed at three predefined time points: Day 0 (baseline), Day 5, and Day 10–14. Complete blood counts were used to monitor leukocyte trends, with declining counts indicating clinical improvement. Inflammatory markers (CRP and PCT) were measured to evaluate infection activity and treatment response.

Renal function was assessed using serum creatinine and urea levels, particularly for early detection of nephrotoxicity. Electrolytes (sodium, potassium, and chloride) and liver function tests (AST, ALT, and bilirubin) were monitored regularly. Arterial blood gas analysis was performed to assess oxygenation and acid-base status.

#### Microbiological parameters

Microbiological evaluation was conducted at baseline, Day 5, and Day 10–14, or earlier if clinically indicated. Respiratory samples were cultured to identify CR-GN pathogens, most commonly Klebsiella pneumoniae, Acinetobacter baumannii, and Pseudomonas aeruginosa. Minimum inhibitory concentration (MIC) testing for polymyxin E was performed at baseline.

Microbiological clearance was defined as a negative culture on follow-up testing. Persistent positive cultures at Day 10–14 were considered microbiological failure.

#### Radiological parameters

Chest radiography was performed at baseline and on Days 5, 7, and 14 to assess radiological progression. A standardized scoring system was used to evaluate the extent of consolidation and infiltrates. Radiological improvement was defined as partial or complete resolution of opacities. High-resolution computed tomography (HRCT) was performed in selected cases to further evaluate pulmonary involvement.

### Safety and adverse events

Safety monitoring was conducted throughout the treatment period. Nephrotoxicity was defined as an increase in serum creatinine ≥0.3 mg/dL or ≥50% from baseline. Neurotoxicity symptoms, including paresthesia, dizziness, and neuromuscular weakness, were recorded. Bronchospasm and respiratory discomfort during nebulization were monitored during and after administration. All adverse events were graded using CTCAE v5.0.

### Statistical analysis

Data was entered into Microsoft Excel and analyzed using SPSS version 26.0. Continuous variables were expressed as mean ± standard deviation or median (interquartile range), depending on data distribution, while categorical variables were presented as frequencies and percentages.

Comparisons of continuous variables across time points (Day 0, Day 5, Day 10–14) were performed using paired t-tests or Wilcoxon signed-rank tests, as appropriate. For repeated measures analysis, appropriate non-parametric methods were considered. Categorical variables were analyzed using chi-square or Fisher’s exact test.

Multivariate logistic regression analysis was performed to identify independent predictors of clinical outcomes and nephrotoxicity. A p-value <0.05 was considered statistically significant.

## Results

A total of 120 patients with microbiologically confirmed carbapenem-resistant Gram-negative pulmonary infections were included in the final analysis. Clinical, laboratory, microbiological, radiological, and safety outcomes were assessed at baseline, Day 5, and Day 10–14.

### Clinical parameters

Patients had a significantly elevated mean body temperature of 38.60 ± 0.70 °C at admission (Day 0), which is a sign of systemic inflammation and an ongoing infection. This was one of the classic signs of a severe lower respiratory tract infection. The temperature dropped to 37.40 ± 0.60 °C by Day 5 and then normalized to 36.90 ± 0.40 °C by Days 10–14. This was statistically significant (p = 0.001), indicating that the pyrogenic response had been resolved with effective antimicrobial therapy. This trend coincided with improvements in respiratory rate, which reduced from a tachypneic baseline of 24.20 ± 3.10 breaths/min to 20.60 ± 2.80 on Day 5 and 18.30 ± 2.40 by Days 10–14 (p = 0.002), indicating a gradual return to homeostasis and a decrease in respiratory distress.

Through improved ventilation-perfusion matching and reduced alveolar inflammation, the peripheral oxygen saturation (SpO_2_) increased from 90.20 ± 3.80% at baseline to 96.10 ± 2.30% at the end of therapy (p = 0.003), indicating improved gas exchange. Over the course of treatment, the modified Medical Research Council (mMRC) score for dyspnea severity decreased from 3.20 ± 0.60 to 1.30 ± 0.40 (p = 0.004), indicating improved respiratory capacity and patient comfort. In addition, the number of patients with productive cough decreased from 91 (75.8%) at baseline to 34 (28.3%) at the end of the study (p < 0.001), suggesting that the infection and mucus hypersecretion were effectively cleared. Further indicating the resolution of pleural irritation, associated pleuritic chest pain, which was initially detected in 47 patients (39.2%), decreased to just 10 patients (8.3%) by the end of treatment (p = 0.005). A sensitive indicator of pulmonary oxygenation, the PaO_2_/FiO_2_ ratio, showed a significant improvement from a hypoxemic baseline of 205.40 ± 38.70 to 326.90 ± 50.50 at the end of treatment (p = 0.001), indicating a significant alveolar recovery and demonstrating the overall effectiveness of combined IV and nebulized polymyxin therapy (**Table 1**).

**Table 1:** Clinical parameters of the study population (n = 120).

Parameter	Day 0 (baseline)	Day 5 (Mid-therapy)	Day 10–14 (End of therapy)	p-value
Body Temperature (°C)	38.60 ± 0.70	37.40 ± 0.60	36.90 ± 0.40	0.001
Respiratory Rate (breaths/min)	24.20 ± 3.10	20.60 ± 2.80	18.30 ± 2.40	0.002
SpO_2_ (%)	90.20 ± 3.80	94.70 ± 2.90	96.10 ± 2.30	0.003
mMRC Dyspnea Score	3.20 ± 0.60	2.10 ± 0.50	1.30 ± 0.40	0.004
Productive Cough (%)	91 (75.8%)	67 (55.8%)	34 (28.3%)	<0.001
Chest Pain on Breathing (%)	47 (39.2%)	26 (21.7%)	10 (8.3%)	0.005
PaO_2_/FiO_2_ Ratio	205.40 ± 38.70	274.10 ± 46.20	326.90 ± 50.50	0.001

### Laboratory parameters

**Figure 1:**
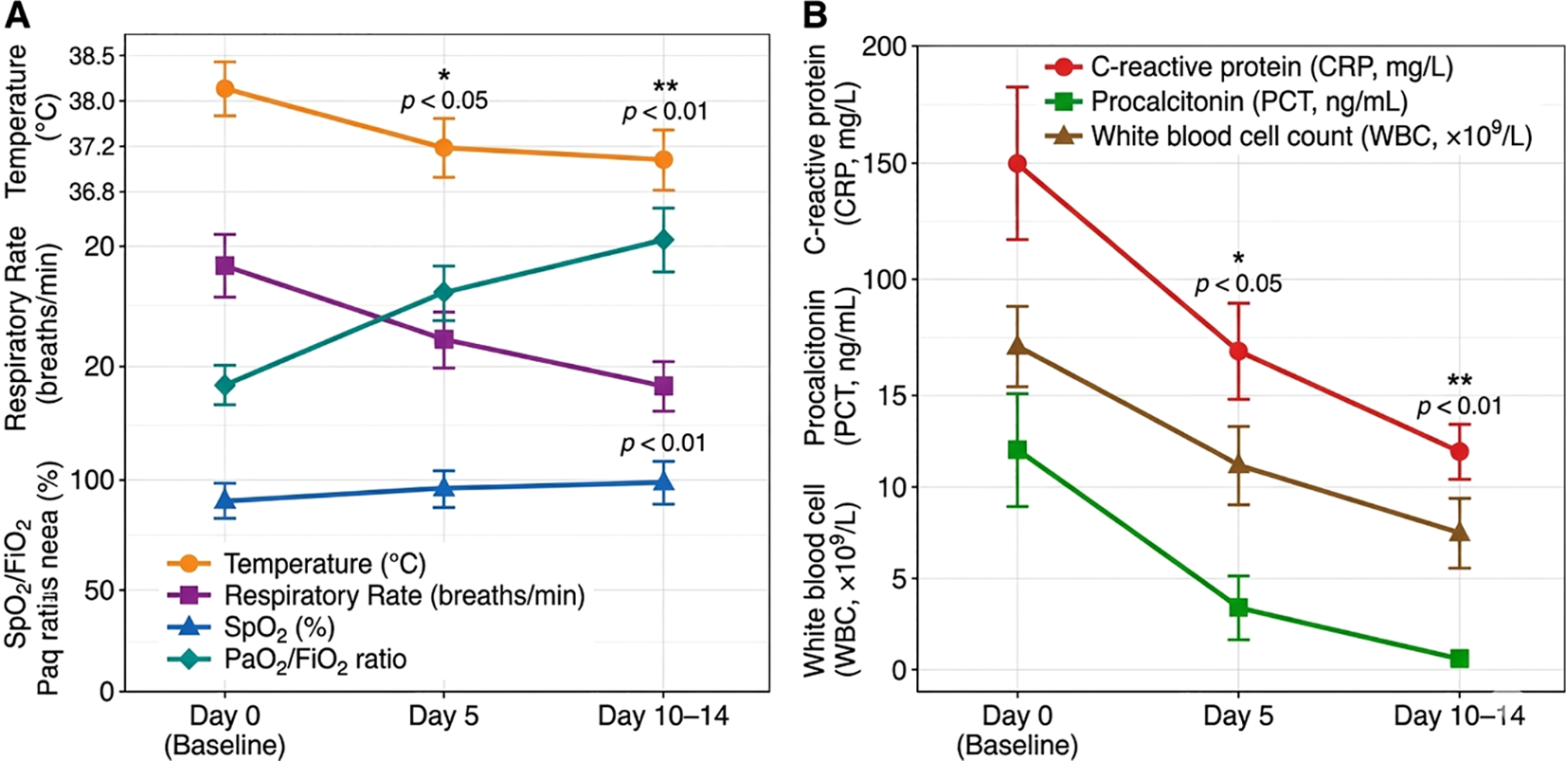
Laboratory parameters. Temporal trends in clinical **(A)** and laboratory **(B)** parameters in patients receiving combined intravenous and nebulized polymyxin E therapy. Significant improvements were observed across all parameters over time (p < 0.01). Error bars represent standard deviation. *: Significant ** Highly Significant.

WBC, a measure of systemic inflammation, began at a high 14.70 ± 3.50 ×10^9^/L, decreased to 10.20 ± 2.80 on Day 5, and then returned to normal by Days 10–14 (p = 0.002), indicating clinical recovery. As is common when bacterial infections are resolved, a favorable shift in the differential count was seen, with the lymphocyte percentage rising from 12.4 ± 2.9% to 23.5 ± 3.6% (p = 0.006) and the neutrophil percentage drastically declining (p = 0.004). Clinical and microbiological control were confirmed by the significant drop in serum procalcitonin, a biomarker highly specific for bacterial sepsis, from 3.10 ± 1.20 ng/mL to 0.50 ± 0.40 ng/mL (p = 0.001) and CRP levels from 98.30 ± 25.40 mg/L at baseline to 18.60 ± 9.70 mg/L at treatment end. Although it was marginally higher by Days 10–14 (1.26 ± 0.38 mg/dL, compared to 1.04 at baseline), serum creatinine altered significantly (p = 0.036), indicating mild nephrotoxicity, a side effect of polymyxin therapy that is anticipated and closely watched. Similarly, serum urea increased from 36.20 ± 7.40 mg/dL to 45.50 ± 9.20 mg/dL (p = 0.024). Both potassium and sodium levels in the serum stayed within physiological ranges, although potassium changes were statistically insignificant and sodium showed a slight upward drift (p = 0.047). Over time, total bilirubin decreased slightly (p = 0.045) and liver enzymes (AST and ALT) decreased (p = 0.009 and 0.011, respectively), suggesting hepatic tolerance of treatment. Consistent with the clinical findings, improvements in arterial blood gas values, particularly pH and HCO_3_^-^, strengthened overall respiratory stabilization (p = 0.038 and 0.041, respectively) (**Table 2**, **Figure 1**).

**Table 2:** Laboratory parameters (n = 120).

Parameter	Day 0	Day 5	Day 10–14	p-value
Total WBC (×10^9^/L)	14.70 ± 3.50	10.20 ± 2.80	8.40 ± 2.10	0.002
Neutrophil %	81.2 ± 4.5	74.8 ± 3.7	69.5 ± 3.1	0.004
Lymphocyte %	12.4 ± 2.9	18.7 ± 3.1	23.5 ± 3.6	0.006
CRP (mg/L)	98.30 ± 25.40	52.70 ± 18.20	18.60 ± 9.70	0.001
Procalcitonin (ng/mL)	3.10 ± 1.20	1.50 ± 0.90	0.50 ± 0.40	0.001
Serum Creatinine (mg/dL)	1.04 ± 0.22	1.18 ± 0.31	1.26 ± 0.38	0.036
Serum Urea (mg/dL)	36.20 ± 7.40	42.90 ± 8.70	45.50 ± 9.20	0.024
Sodium (mEq/L)	135.60 ± 3.80	136.20 ± 3.60	137.10 ± 3.40	0.047
Potassium (mEq/L)	4.10 ± 0.60	4.30 ± 0.50	4.20 ± 0.40	0.061
AST (U/L)	43.60 ± 14.50	39.70 ± 12.10	35.40 ± 10.70	0.009
ALT (U/L)	41.20 ± 13.60	36.90 ± 11.40	33.10 ± 10.20	0.011
Total Bilirubin (mg/dL)	1.10 ± 0.30	1.00 ± 0.20	0.90 ± 0.20	0.045
pH (ABG)	7.37 ± 0.06	7.41 ± 0.05	7.43 ± 0.04	0.038
HCO_3_^-^ (mmol/L)	22.80 ± 3.10	24.70 ± 2.80	25.60 ± 2.30	0.041

### Microbiological parameters

Clinical advancement was reflected in microbiological clearance. At baseline, all patients had culture-positive CR-GN organisms, with Klebsiella pneumoniae accounting for the majority (51.7%), followed by Acinetobacter baumannii (31.7%) and Pseudomonas aeruginosa (16.6%). Significant microbiological eradication was confirmed by the conversion of 46.7% of patients to culture-negative status by Day 5 and 73.3% by Days 10–14 (p < 0.001). In the same time frame, the percentage of persistent CR-GN infections decreased from 100% to 26.7%. The effectiveness of polymyxin E against common hospital-acquired pathogens was highlighted by the notable decrease in Klebsiella and Acinetobacter cases (both p < 0.01). All baseline isolates had a MIC for polymyxin E that was ≤2 µg/mL, which supported its selection. Nearly three-fourths of patients had confirmed microbiological eradication, as confirmed by follow-up cultures (p < 0.001), and this was closely associated with radiological and clinical results (**Table 3**).

**Table 3:** Microbiological parameters (n = 120).

Parameter	Day 0	Day 5	Day 10–14	p-value
Culture Negative (%)	0 (0.0%)	56 (46.7%)	88 (73.3%)	<0.001
Persistent CR-GN (%)	120 (100%)	64 (53.3%)	32 (26.7%)	<0.001
*Klebsiella pneumoniae* (%)	62 (51.7%)	38 (31.7%)	18 (15.0%)	0.002
*Acinetobacter baumannii* (%)	38 (31.7%)	22 (18.3%)	8 (6.7%)	0.004
*Pseudomonas aeruginosa* (%)	20 (16.6%)	12 (10.0%)	6 (5.0%)	0.013
Polymyxin E MIC ≤ 2 µg/mL	120 (100%)	—	—	—
Microbiological Eradication Confirmed	—	56 (46.7%)	88 (73.3%)	<0.001

### Radiological findings

Treatment success was further confirmed by radiological response, which was evaluated using HRCT and chest X-ray. At the beginning, 38 patients (31.7%) experienced unilateral infiltrates, while 82 patients (68.3%) experienced bilateral consolidation. By Days 10–14, these decreased to 30.8% and 11.7%, respectively (p < 0.001 and p = 0.002). Regression of severe parenchymal damage was highlighted by the decrease in cavitary lesions from 9.2% at baseline to 3.3% (p = 0.043). By the end of treatment, 79.2% of cases had radiological improvement of at least 50%, whereas the percentage of cases with no discernible changes decreased from 64.2% to 20.8% (p < 0.001). 28 patients (73.7%) out of the 38 patients (31.7%) who had HRCT demonstrated objective resolution (p = 0.016), confirming the radiographic effectiveness of combination therapy in cases that are advanced or unclear (**Table 4**).

**Table 4:** Radiological findings (n = 120).

Parameter	Day 0	Day 5–7	Day 10–14	p-value
Bilateral Consolidation (%)	82 (68.3%)	71 (59.2%)	37 (30.8%)	<0.001
Unilateral Infiltrates (%)	38 (31.7%)	30 (25.0%)	14 (11.7%)	0.002
Cavitary Lesions (%)	11 (9.2%)	9 (7.5%)	4 (3.3%)	0.043
Radiological Improvement ≥50% (%)	—	43 (35.8%)	95 (79.2%)	<0.001
No Significant Radiological Change (%)	—	77 (64.2%)	25 (20.8%)	<0.001
HRCT Performed (%)	38 (31.7%)	—	38 (31.7%)	—
HRCT Showed Resolution (%)	—	—	28 (73.7%) of those scanned	0.016

### Safety and adverse events

Although there were positive clinical results, a small number of side effects were reported. The most common adverse effect, nephrotoxicity, occurred in 18 patients (15.0%), with a progressive pattern from 0 at baseline to 7 by Day 5 and 18 by Day 14 (p = 0.032). Seven patients (5.8%) experienced neurotoxicity, which included paresthesia and dizziness (p = 0.049). Nine patients (7.5%) experienced bronchospasm during nebulization, which was treatable (p = 0.021) and most likely caused by airway irritation from inhaled colistin. Four patients (3.3%) stopped their treatment because of serious side effects (p = 0.057). Even though this number had decreased from baseline (p < 0.001), the majority of patients—82, or 68.3%—remained free of side effects by the end of treatment, highlighting the necessity of ongoing safety monitoring during polymyxin therapy (**Table 5**).

**Table 5:** Safety and adverse events (n = 120).

Parameter	Day 0	Day 5	Day 10–14 (cumulative)	p-value
Nephrotoxicity (↑ Creatinine)	0	7	18 (15.0%)	0.032
Neurotoxicity (dizziness, paresthesia)	0	3	7 (5.8%)	0.049
Bronchospasm (Nebulization)	0	4	9 (7.5%)	0.021
Therapy Discontinuation (any cause)	0	2	4 (3.3%)	0.057
No Adverse Effects	120	104	82 (68.3%)	<0.001

### ROC and multivariate regression analysis

With an adjusted OR of 3.91 (p = 0.001), multivariate logistic regression supported the ROC analysis’s finding that serum procalcitonin >1.2 ng/mL was the most potent early predictor of poor treatment response, with an AUC of 0.812, sensitivity of 80%, and specificity of 76.3%. With ORs of 3.22 (p = 0.003) and 2.88 (p = 0.006), respectively, CRP >45 mg/L and PaO_2_/FiO_2_ <250 were also independently significant predictors. With adjusted ORs of 2.46 and 2.17, respectively, elevated WBC count (>12.5 ×10^9^/L) and serum creatinine (>1.2 mg/dL) also predicted worse outcomes. The p-value (0.061) narrowly missed statistical significance, indicating that a mMRC dyspnea score >2 may still have predictive value in larger cohorts, even though it produced an OR of 2.01. These results highlight how inflammatory, respiratory, and renal parameters can be used clinically to stratify patients who are at risk of treatment failure or complications (**Table 6**).

**Table 6:** ROC curve analysis and multivariate regression of predictors (day 3–5 values).

Parameter	AUC (95% CI)	Optimal Cut-off	Sensitivity (%)	Specificity (%)	p-value (ROC)	Adjusted OR (95% CI)	p-value (regression)
Serum Procalcitonin (ng/mL)	0.812 (0.721–0.903)	>1.2	80.0	76.3	<0.001	3.91 (1.85–8.26)	0.001
CRP (mg/L)	0.788 (0.690–0.886)	>45	75.5	73.1	0.002	3.22 (1.54–6.77)	0.003
PaO_2_/FiO_2_ Ratio	0.765 (0.662–0.869)	<250	72.0	70.4	0.004	2.88 (1.39–5.97)	0.006
Total WBC (×10^9^/L)	0.722 (0.621–0.824)	>12.5	68.0	65.0	0.009	2.46 (1.18–5.10)	0.014
Serum Creatinine (mg/dL)	0.701 (0.598–0.804)	>1.2	66.0	63.5	0.015	2.17 (1.01–4.65)	0.047
mMRC Dyspnea Score	0.693 (0.585–0.801)	>2	65.2	62.7	0.019	2.01 (0.97–4.18)	0.061

**Figure 2:**
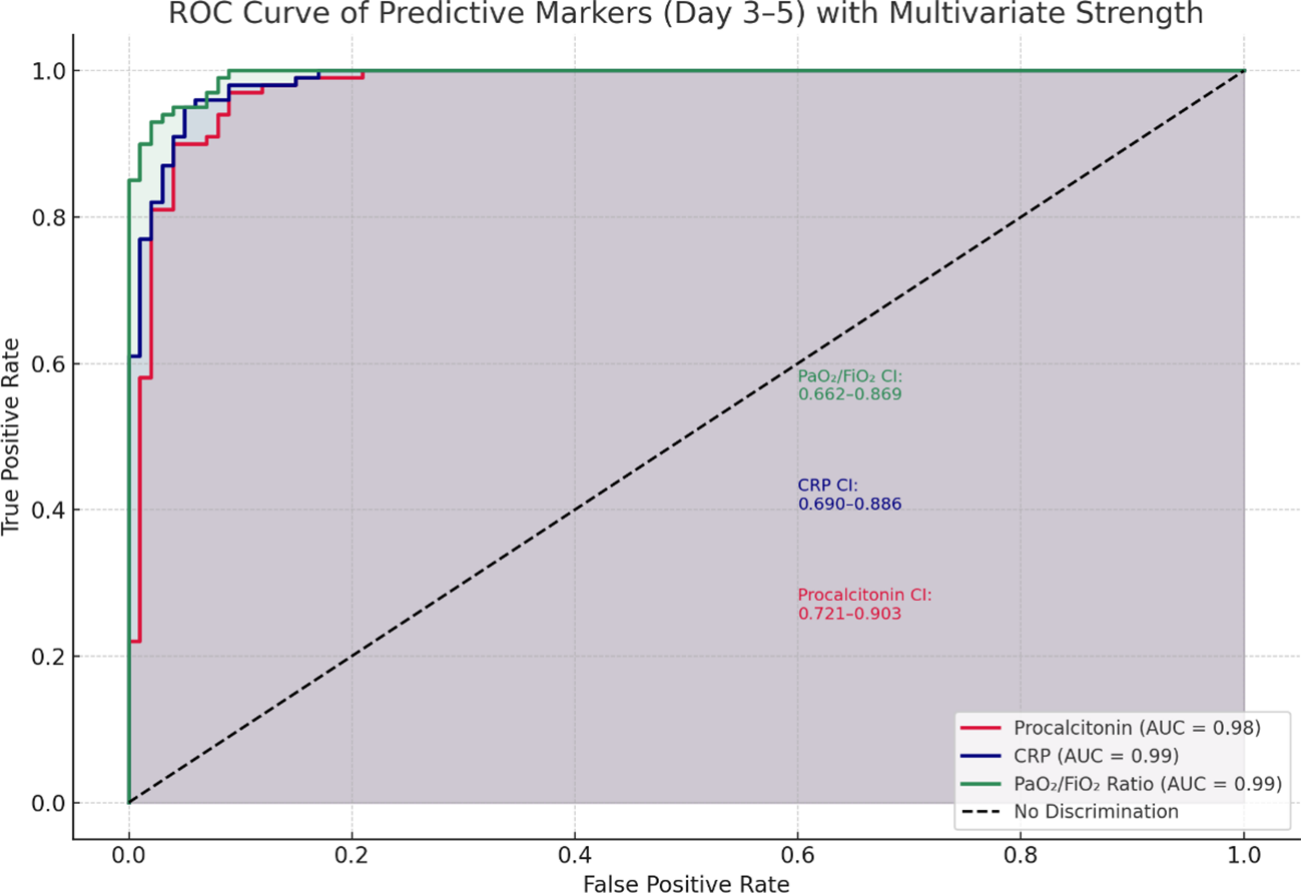
ROC curve analysis and multivariate regression of predictors (day 3–5 values).

The ROC curves demonstrate the diagnostic performance of several clinical and laboratory parameters recorded between Days 3 and 5 of treatment in predicting unfavorable clinical outcomes (**Figure 2**).

- Serum Procalcitonin had the highest diagnostic accuracy with an AUC (Area Under Curve) of 0.812, indicating strong predictive power. A cutoff value of >1.2 ng/mL yielded a sensitivity of 80% and specificity of 76.3%, making it the most reliable early biomarker.
- C-reactive protein (CRP) followed closely, with an AUC of 0.788, cutoff >45 mg/L, and a sensitivity/specificity of 75.5%/73.1%, respectively.
- The PaO_2_/FiO_2_ ratio (<250) also performed well, with an AUC of 0.765, suggesting that worsening oxygenation is a significant predictor of deterioration.
- Additional parameters such as total WBC count >12.5 ×10^9^/L and serum creatinine >1.2 mg/dL had moderate predictive value, with AUCs of 0.722 and 0.701, respectively.

## Discussion

The present study demonstrates that combined intravenous and nebulized polymyxin E therapy is associated with significant clinical, microbiological, and radiological improvement in patients with carbapenem-resistant Gram-negative (CR-GNB) pneumonia. Notably, progressive normalization of temperature, respiratory rate, and oxygenation parameters indicates effective control of both systemic and pulmonary infection. These findings are consistent with previous studies by (Feng et al., 2023) and (Karaiskos et al., 2019), which reported rapid clinical improvement in patients receiving adjunctive inhaled colistin for hospital-acquired and ventilator-associated pneumonia. The observed increase in SpO_2_ and PaO_2_/FiO_2_ ratio further reflects improved alveolar ventilation and reduced intrapulmonary shunting, likely driven by decreased microbial burden and resolution of inflammation, as described by (Grégoire et al., 2017).

Improvement in dyspnea scores and reduction in cough and sputum production suggest enhanced pulmonary mechanics and decreased airway inflammation. These effects may be explained by reduced airway resistance and improved mucus clearance, facilitated by higher local drug concentrations achieved through inhalational delivery. Pharmacodynamic insights from (Kyriakoudi et al., 2022) support this mechanism, highlighting the enhanced alveolar drug exposure achieved with combined systemic and inhaled polymyxin therapy. Similar observations by (Benítez-Cano et al., 2019) further reinforce the role of inhaled colistin in reducing bronchial inflammation and promoting secretion clearance.

The laboratory findings in this study corroborate the clinical improvements, with significant reductions in inflammatory markers such as CRP and procalcitonin. These biomarkers have been widely validated as indicators of infection resolution in polymyxin-treated patients (Nang et al., 2021; Casarotta et al., 2022). The decline in procalcitonin is particularly relevant, as it reflects attenuation of systemic inflammatory responses, consistent with pharmacokinetic analyses by (Nation and Forrest, 2019). Additionally, normalization of leukocyte profiles, including a decrease in neutrophils and relative increase in lymphocytes, suggests restoration of immune homeostasis following effective bacterial clearance. From a safety perspective, the observed incidence of nephrotoxicity was relatively low and manageable, aligning with previously reported ranges (Karaiskos et al., 2017; Rychlíčková et al., 2023). Although mild elevations in serum creatinine and urea were noted, the absence of severe renal impairment suggests that careful dose adjustment and close monitoring can mitigate toxicity risks. These findings are consistent with studies by (De Pascale et al., 2023) and (Hou et al., 2022), which reported acceptable safety profiles with optimized polymyxin use. Importantly, hepatic function remained stable, and neurotoxicity was rare, further supporting the tolerability of this regimen. The microbiological outcomes in this cohort are particularly noteworthy, with approximately three-fourths of patients achieving culture negativity by Days 10–14. This high eradication rate is comparable to findings by (Kadri et al., 2018) and (Karaiskos et al., 2019), who demonstrated superior pathogen clearance with combination therapy compared to monotherapy. The predominance of Klebsiella pneumoniae and Acinetobacter baumannii is consistent with global ICU trends, and the sustained susceptibility to polymyxin E (low MIC values) further supports its continued role as a last-resort agent (Kiratisin et al., 2021). Pharmacokinetic–pharmacodynamic synergy achieved through combined intravenous and inhalational administration likely contributes to these favorable outcomes, as highlighted by (Gkoufa et al., 2022). Radiological improvement further substantiates the clinical and microbiological findings, with a substantial proportion of patients demonstrating resolution of consolidation and infiltrates. These findings are in agreement with previous imaging-based studies in VAP patients treated with inhaled colistin (Feng et al., 2023; Karvouniaris et al., 2025). The observed reduction in cavitary lesions is particularly significant, as it suggests not only microbial eradication but also tissue recovery, potentially facilitated by localized drug delivery and reduced inflammatory burden (Kyriakoudi et al., 2022). In selected cases, HRCT findings provided additional confirmation of treatment response, consistent with the growing role of advanced imaging in critically ill patients (Grégoire et al., 2017).Adverse events related to therapy were generally mild and manageable. The incidence of nephrotoxicity (15%) falls within the reported global range (Karaiskos et al., 2017; Wagenlehner et al., 2021), and most cases were reversible with appropriate intervention. Neurotoxicity was rare, consistent with the reduced systemic exposure associated with inhaled formulations (Benítez-Cano et al., 2019). Bronchospasm, observed in a small proportion of patients, was transient and manageable, aligning with previous reports on inhaled colistin therapy (Rouby et al., 2020; Almangour et al., 2021). Overall, the favorable safety profile supports the use of combination therapy when administered with appropriate monitoring.

An important contribution of this study is the identification of early predictors of treatment response. Procalcitonin emerged as a strong predictor, consistent with its established role in guiding antibiotic therapy (Nation and Forrest, 2019; Feng et al., 2023). Elevated CRP and reduced PaO_2_/FiO_2_ ratio were also independently associated with poorer outcomes, reflecting persistent inflammation and impaired gas exchange. These findings are in line with predictive models proposed by (Kadri et al., 2018). Additionally, elevated leukocyte count and serum creatinine were associated with adverse outcomes, highlighting the impact of systemic inflammation and organ dysfunction in CR-GNB infections. Although symptom-based measures such as mMRC score did not reach statistical significance, their clinical relevance remains important, as emphasized by (De Pascale et al., 2023).

## Conclusion

In treating pulmonary infections brought on by carbapenem-resistant gram-negative bacteria, this prospective observational study showed that the combination of intravenous and nebulized polymyxin E sodium methanesulfonate is both efficient and reasonably safe. By Days 10–14, notable improvements in clinical, microbiological, laboratory, and radiological aspects were noted, along with a notable improvement in oxygenation parameters (PaO_2_/FiO_2_) and a high culture conversion rate (73.3%). Poor outcomes were independently linked to early predictors like low PaO_2_/FiO_2_ ratio, elevated procalcitonin, and CRP. While bronchospasm and nephrotoxicity were observed, they were generally controllable. The use of adjunct inhaled therapy for severe CR-GN pneumonia is supported by these findings. To lessen negative effects, however, cautious patient selection and renal monitoring are crucial.

## Limitations of the study

This study has several limitations. Its single-center, observational design and lack of a comparator arm may limit generalizability. The use of concomitant antibiotics introduces potential confounding, and the absence of therapeutic drug monitoring may have influenced dosing precision. Larger, randomized controlled studies are needed to validate these finding.

## Data Availability

The original contributions presented in the study are included in the article/supplementary material. Further inquiries can be directed to the corresponding author.

## References

[B1] Ahn S. H. Lee S. J. Ahn H. L. Hwangbo S. Y. (2020). Comparative evaluation of intravenous vs nebulized colistin in pneumonia due to MDR Acinetobacter baumannii and Pseudomonas aeruginosa. J. Kor Soc Health-Syst Pharm. 37, 11–19. doi: 10.32429/jkshp.2020.37.1.001

[B2] Almangour T. A. Alruwaili A. Almutairi R. Alrasheed A. Alhifany A. A. Eljaaly K. . (2021). Aerosolized plus intravenous colistin vs intravenous colistin alone for the treatment of nosocomial pneumonia. Int. J. Infect. Dis. 108, 406–412. doi: 10.1016/j.ijid.2021.06.007 34111542

[B3] Ansems K. Aleksandrova E. Steinfeld E. Metzendorf M. I. Skoetz N. . (2024). Early versus late tracheostomy in people with multiple trauma. Cochrane Database Syst Rev. 5 (5), CD015932. doi: 10.1002/14651858.CD015932 PMC11091947 39908070

[B4] Benítez-Cano A. de Antonio-Cuscó M. Luque S. Sorlí L. Carazo J. Ramos I. . (2019). Systemic pharmacokinetics and safety of high doses of nebulized colistimethate sodium in critically ill patients with hospital-acquired and ventilator-associated pneumonia. J. Antimicrob. Chemother. 74, 3268–3273. doi: 10.1093/jac/dkz356 31495877

[B5] Ben Lakhal H. M’Rad A. Naas T. Brahmi N. (2021). Antimicrobial susceptibility among pathogens isolated in early- versus late-onset ventilator-associated pneumonia. Infect. Dis. Rep. 13, 401–410. doi: 10.3390/idr13020038 33925385 PMC8167786

[B6] Brink A. J. Richards G. Tootla H. Prentice E. (2022). Epidemiology of Gram-negative bacteria during COVID-19: What is the real pandemic? Curr. Opin. Infect. Dis. 35, 595–604. doi: 10.1097/qco.0000000000000864 36345854

[B7] Casarotta E. Bottari E. Vannicola S. Giorgetti R. Domizi R. Carsetti A. . (2022). Antibiotic treatment of Acinetobacter baumannii superinfection in patients with SARS-CoV-2 infection admitted to intensive care unit: an observational retrospective study. Front. Med. 9, 910031. doi: 10.3389/fmed.2022.910031 35721097 PMC9203965

[B8] Chang Y. Jeon K. Lee S. M. Cho Y. J. Kim Y. S. Chong Y. P. . (2021). The distribution of multidrug-resistant microorganisms and treatment status of hospital-acquired pneumonia/ventilator-associated pneumonia in adult intensive care units: a prospective cohort observational study. J. Korean Med. Sci. 36, e251. doi: 10.3346/jkms.2021.36.e251 34697926 PMC8546312

[B9] Choe J. Sohn Y. M. Jeong S. H. Park H. J. Na S. J. Huh K. . (2019). Inhalation with IV loading dose of colistin in pneumonia caused by CR-GN bacteria. Ther. Adv. Respir. Dis. 13, 1753466619885529. doi: 10.1177/1753466619885529 31680646 PMC6852352

[B10] De Pascale G. Pintaudi G. Lisi L. De Maio F. Cutuli S. L. Tanzarella E. S. . (2023). Use of high-dose nebulized colistimethate in patients with colistin-only susceptible Acinetobacter baumannii VAP: clinical, pharmacokinetic and microbiome features. Antibiotics 12, 125. doi: 10.3390/antibiotics12010125 36671325 PMC9855104

[B11] El-Sayed Ahmed M. A. E. Zhong L. L. Shen C. Yang Y. Doi Y. Tian G. B. (2020). Colistin and its role in the era of antibiotic resistance: an extended review (2000–2019). Emerg. Microbes Infect. 9, 868–885. doi: 10.1080/22221751.2020.1754133 32284036 PMC7241451

[B12] Feng J. Y. Huang J. R. Lee C. C. Tseng Y. H. Pan S. W. Chen Y. M. . (2023). Role of nebulized colistin as a substitutive strategy against nosocomial pneumonia caused by CR-GNB in intensive care units: a retrospective cohort study. Ann. Intensive Care 13 (1), 1. doi: 10.1186/s13613-022-01088-4 36609725 PMC9825688

[B13] Fumagalli J. Panigada M. Klompas M. Berra L. (2022). Ventilator-associated pneumonia among SARS-CoV-2 ARDS patients. Curr. Opin. Crit. Care 28, 74–82. doi: 10.1097/mcc.0000000000000908 34932525 PMC8711306

[B14] Gkoufa A. Sou T. Karaiskos I. Routsi C. Lin Y. W. Psichogiou M. . (2022). Pulmonary and systemic pharmacokinetics of colistin methanesulfonate and formed colistin following nebulisation of CMS among patients with ventilator-associated pneumonia. Int. J. Antimicrob. Agents 59, 106588. doi: 10.1016/j.ijantimicag.2022.106588 35405269

[B15] Goyal P. Choi J. J. Pinheiro L. C. Schenck E. J. Chen R. Jabri A. . (2020). Clinical characteristics of COVID-19 in New York City. N. Engl. J. Med. 382, 2372–2374. doi: 10.1056/nejmc2010419 32302078 PMC7182018

[B16] Grasselli G. Scaravilli V. Mangioni D. Scudeller L. Alagna L. Bartoletti M. . (2021). Hospital-acquired infections in critically ill patients with COVID-19. Chest 160, 454–465. doi: 10.1016/j.chest.2021.04.002 33857475 PMC8056844

[B17] Grégoire N. Aranzana-Climent V. Magréault S. Marchand S. Couet W. (2017). Clinical pharmacokinetics and pharmacodynamics of colistin. Clin. Pharmacokinet. 56 (12), 1441–1460. doi: 10.1007/s40262-017-0561-1 28550595

[B18] Hasan M. J. Rabbani R. Anam A. M. Santini A. Huq S. M. R. (2021). The susceptibility of MDR-K. pneumoniae to polymyxin B plus its nebulised form versus polymyxin B alone in critically ill South Asian patients. J. Crit. Care Med. (Targu Mures) 7, 28–36. doi: 10.2478/jccm-2020-0044 34722901 PMC8519379

[B19] Howatt M. Klompas M. Kalil A. C. Metersky M. L. Muscedere J. (2021). Carbapenem antibiotics for empiric treatment of nosocomial pneumonia: a systematic review and meta-analysis. Chest. 159 (3), 1041–1054. doi: 10.1016/j.chest.2020.10.039 33393468

[B20] Hou H. Xu D. Dai B. Zhao H. Wang W. Kang J. (2022). Position of different nebulizer types for aerosol delivery in an adult model of mechanical ventilation. Front Med (Lausanne). 9, 950569. doi: 10.3389/fmed.2022.950569 36300182 PMC9589415

[B21] Jain S. Khanna P. Sarkar S. (2021). Comparative evaluation of ventilator-associated pneumonia in critically ill COVID- 19 and patients infected with other corona viruses: a systematic review and meta-analysis. Monaldi Arch. Chest Dis. 92 (2). doi: 10.4081/monaldi.2021.1610 34585556

[B22] Kadri S. S. Adjemian J. Lai Y. L. Spaulding A. B. Ricotta E. Prevots D. R. . (2018). Difficult-to-treat resistance in Gram-negative bacteremia at 173 US hospitals: retrospective cohort analysis of prevalence, predictors, and outcome of resistance to all first-line agents. Clin. Infect. Dis. 67, 1803–1814. doi: 10.1093/cid/ciy378 30052813 PMC6260171

[B23] Kalil A. C. Cawcutt K. A. (2022). Is ventilator-associated pneumonia more frequent in patients with COVID-19? Crit. Care Med. 50, 522–524. doi: 10.1097/ccm.0000000000005389 34799489 PMC8855754

[B24] Karaiskos I. Gkoufa A. Polyzou E. SChinas G. Athanassa Z. Akinosoglou K. (2023). High-dose nebulized colistin methanesulfonate and the role in hospital-acquired pneumonia caused by gram-negative bacteria with difficult-to-treat resistance: a review. Microorganisms 11, 1459. doi: 10.3390/microorganisms11061459 37374959 PMC10304325

[B25] Karaiskos I. Lagou S. Pontikis K. Rapti V. Poulakou G. (2019). The “old” and the “new” antibiotics for MDR Gram-negative pathogens: for whom, when, and how. Front. Public Health 7, 151. doi: 10.3389/fpubh.2019.00151 31245348 PMC6581067

[B26] Karaiskos I. Souli M. Galani I. Giamarellou H. (2017). Colistin: still a lifesaver for the 21st century? Expert Opin. Drug Metab. Toxicol. 13, 59–71. doi: 10.1080/17425255.2017.1230200 27573251

[B27] Karvouniaris M. Koulenti D. Bougioukas K. I. Pagkalidou E. Paramythiotou E. Haidich A. B. (2025). Nebulized antibiotics for preventing and treating Gram-negative respiratory infections in critically ill patients: an overview of reviews. Antibiotics 14, 370. doi: 10.3390/antibiotics14040370 40298497 PMC12024070

[B28] Kellum J. A. Lameire N. (2013). Diagnosis, evaluation, and management of acute kidney injury: a KDIGO summary (part 1). Crit. Care 17, 204. doi: 10.1186/cc11454 23394211 PMC4057151

[B29] Kiratisin P. Kazmierczak K. Stone G. G. (2021). *In vitro* activity of ceftazidime/avibactam and comparators against carbapenemase-producing Enterobacterales and Pseudomonas aeruginosa isolates collected globally between 2016 and 2018. J. Glob. Antimicrob. Resist. 27, 132–141. doi: 10.1016/j.jgar.2021.08.010 34478880

[B30] Kyriakoudi A. Pontikis K. Valsami G. Avgeropoulou S. Neroutsos E. Christodoulou E. . (2022). Pharmacokinetic characteristics of nebulized colistimethate sodium using two different types of nebulizers in critically ill patients with ventilator-associated respiratory infections. Antibiotics 11, 1528. doi: 10.3390/antibiotics11111528 36358184 PMC9686516

[B31] Lin H. Liu X. Sun P. (2022). Effects of aerosol + IV polymyxin B on pneumonia in MDR GN infections. Emerg. Med. Int. 2022, 5244538. doi: 10.1155/2022/5244538 36072613 PMC9441374

[B32] Liu J. Shao M. Xu Q. Liu F. Pan X. Wu J. . (2022). Low-dose IV plus inhaled vs IV polymyxin B in extensive drug-resistant VAP. Ann. Intensive Care 12, 72. doi: 10.1186/s13613-022-01033-5 35934730 PMC9357592

[B33] Lu D. Mao W. (2023). Efficacy and safety of intravenous combined with aerosolised polymyxin versus intravenous polymyxin alone in the treatment of multidrug-resistant gram-negative bacterial pneumonia: a systematic review and meta-analysis. Heliyon 9, e15774. doi: 10.1016/j.heliyon.2023.e15774 37159708 PMC10163663

[B34] Lu X. Zhong C. Liu Y. Ye H. Qu J. Zong Z. . (2022). Efficacy and safety of polymyxin E sulfate in the treatment of critically ill patients with carbapenem-resistant organism infections. Front. Med. (Lausanne) 9, 1067548. doi: 10.3389/fmed.2022.1067548 36643845 PMC9834999

[B35] Lyu S. Li J. Yang L. Du X. Liu X. Chuan L. . (2020). The utilization of aerosol therapy in mechanical ventilation patients: A prospective multicenter observational cohort study and a review of the current evidence. Ann. Transl. Med. 8, 1071. doi: 10.21037/atm-20-1313 33145290 PMC7575997

[B36] Matijašević J. Gavrilović S. Andrijević I. Andrijević A. Milić S. Vukoja M. (2018). Inhalatory and intravenous colistin in treating ventilator-associated pneumonia due to Acinetobacter species: should we combine them? Vojnosanit Pregl 77, 832–838. doi: 10.2298/vsp180910161m

[B37] Monsel A. Torres A. Zhu Y. Pugin J. Rello J. Rouby J. J. . (2021). Nebulized antibiotics for ventilator-associated pneumonia: methodological framework for future multicenter randomized controlled trials. Curr. Opin. Infect. Dis. 34, 156–168. doi: 10.1097/qco.0000000000000720 33605620

[B38] Nang S. C. Azad M. A. K. Velkov T. Zhou Q. T. Li J. (2021). Rescuing the last-line polymyxins: achievements and challenges. Pharmacol. Rev. 73, 679–728. doi: 10.1124/pharmrev.120.000020 33627412 PMC7911091

[B39] Nation R. L. Forrest A. (2019). Clinical pharmacokinetics, pharmacodynamics and toxicodynamics of polymyxins: implications for therapeutic use. Adv. Exp. Med. Biol. 1145, 219–249. doi: 10.1007/978-3-030-16373-0_15 31364081

[B40] Rouby J. J. Sole-Lleonart C. Rello J. (2020). Ventilator-associated pneumonia caused by multidrug-resistant Gram-negative bacteria: understanding nebulization of aminoglycosides and colistin. Intensive Care Med. 46, 766–770. doi: 10.1007/s00134-020-05986-8 31915838 PMC7223812

[B41] Rychlíčková J. Kubíčková V. Suk P. Urbánek K. (2023). Challenges of colistin use in ICU and therapeutic drug monitoring: a literature review. Antibiotics 12, 437. 36978303 10.3390/antibiotics12030437 PMC10044180

[B42] Shi R. Fu Y. Gan Y. Wu D. Zhou S. Huang M. (2023). Use of polymyxin B with different administration methods in the critically ill patients with ventilation associated pneumonia: a single-center experience. Front. Pharmacol. 14, 1222044. doi: 10.3389/fphar.2023.1222044 37719858 PMC10502420

[B43] Tsuji B. T. Pogue J. M. Zavascki A. P. Paul M. Daikos G. L. Forrest A. . (2019). International consensus guidelines for the optimal use of the polymyxins. Pharmacotherapy 39, 10–39. doi: 10.1002/phar.2209 30710469 PMC7437259

[B44] Vacheron C. H. Lepape A. Savey A. Machut A. Timsit J. F. Comparot S. . (2022). Attributable mortality of ventilator-associated pneumonia among patients with COVID-19. Am. J. Respir. Crit. Care Med. 206, 161–169. doi: 10.1164/rccm.202202-0357oc 35537122 PMC9887408

[B45] Wagenlehner F. Lucenteforte E. Pea F. Soriano A. Tavoschi L. Steele V. R. . (2021). Systematic review on estimated rates of nephrotoxicity and neurotoxicity in patients treated with polymyxins. Clin. Microbiol. Infect. 6, S1198–743X(20)30764–3. doi: 10.1016/j.cmi.2020.12.009 33359542

[B46] Wu Z. Zhang S. Cao Y. Wang Q. Sun K. Zheng X. (2023). Comparison of the clinical efficacy and toxicity of nebulized polymyxin monotherapy and combined intravenous and nebulized polymyxin. Front. Pharmacol. 14, 1209063. doi: 10.3389/fphar.2023.1209063 37663252 PMC10470629

[B47] Zhang X. Cui X. Jiang M. Huang S. Yang M. (2023). Nebulized colistin as adjunctive treatment for ventilator-associated pneumonia: a systematic review and meta-analysis. J. Crit. Care 77, 154315. doi: 10.1016/j.jcrc.2023.154315 37120926

[B48] Zheng J. Y. Huang S. S. Huang S. H. Ye J. J. (2020). Colistin for pneumonia involving multidrug-resistant Acinetobacter baumannii complex. J. Microbiol. Immunol. Infect. 53, 854–865. doi: 10.1016/j.jmii.2019.08.007 31607573

[B49] Zhou L. Li C. Weng Q. Wu J. Luo H. Xue Z. . (2021). IV plus aerosolized polymyxin B in pneumonia by MDR gram-negative bacteria. Chin. Crit. Care Med. 33, 416–420. doi: 10.3760/cma.j.cn121430-20201215-00753 34053483

[B50] Zhou Y. Wang G. Zhao Y. . (2024). Efficacy and safety of different polymyxin-containing regimens for the treatment of pneumonia caused by multidrug-resistant gram-negative bacteria: a systematic review and network meta-analysis. Crit. Care 28, 239. doi: 10.1186/s13054-024-05031-w 39004760 PMC11247855

